# Enhancing antifungal treatment for chronic cavitary pulmonary aspergillosis through the addition of endobronchial valve therapy

**DOI:** 10.1016/j.rmcr.2024.102106

**Published:** 2024-09-17

**Authors:** Thomas Maitre, Juliette Camuset, Morgane Faure, Christophe Cracco, Georgina Maalouf, Yves Allenbach, Matthias Barral, Arnaud Fekkar, Mihaela Giol, Antoine Parrot, Jacques Cadranel

**Affiliations:** a Service de Pneumologie et d’Oncologie Thoracique et Centre Constitutifs Maladies Pulmonaires Rares, Hôpital Tenon, APHP Sorbonne Université, Paris, France; b Centre d’Immunologie et des Maladies Infectieuses (Cimi Paris), INSERM U1135, Sorbonne Université, Paris, France; c Service de Chirurgie Thoracique, Hôpital Tenon, APHP Sorbonne Université, Paris, France; d APHP-6 Sorbonne Université, Site Pitié Salpêtrière, Service de Pneumologie (Département R3S), Paris, France; e Service de Médecine Interne, Hôpital Pitié Salpêtrière, APHP Sorbonne Université, Paris, France; f Service de Radiologie, Hôpital Tenon, APHP Sorbonne Université, Paris, France; g Laboratoire de Parasitologie, Hôpital Pitié Salpêtrière, APHP Sorbonne Université, Paris, France

## Abstract

A 62-year-old male experienced *anti*-MDA5 dermatomyositis with lung involvement, treated with immunosuppressive therapy leading to chronic cavitary pulmonary aspergillosis in left upper lobe. Patient's history was complicated by complete left pneumothorax due to alveolar-pleural fistula occurring because of the rupture of the pulmonary cavitation. Left lung failed to re-expand despite a four-week period of pleural drainage. In addition to antifungal therapy, patient received endobronchial valve therapy in the anterior segmental bronchus of the left upper lobe leading to air leak cessation, left lung re expansion and aspergillosis cavitation closure.

## 1 Introduction

Chronic pulmonary aspergillosis, is a growing disease that leads to significant morbidity and mortality [[1], [2], [3], [4]]. The presence of a lung cavity contributes to the poor prognosis, limited treatment response, and pleural complications associated with chronic pulmonary aspergillosis [[5], [6], [7], [8]]. In this case report, we present a patient with complicated chronic cavitary pulmonary aspergillosis who underwent endobronchial valve therapy as part of their antifungal treatment.

## 2 Case report

**Fig. 1:**
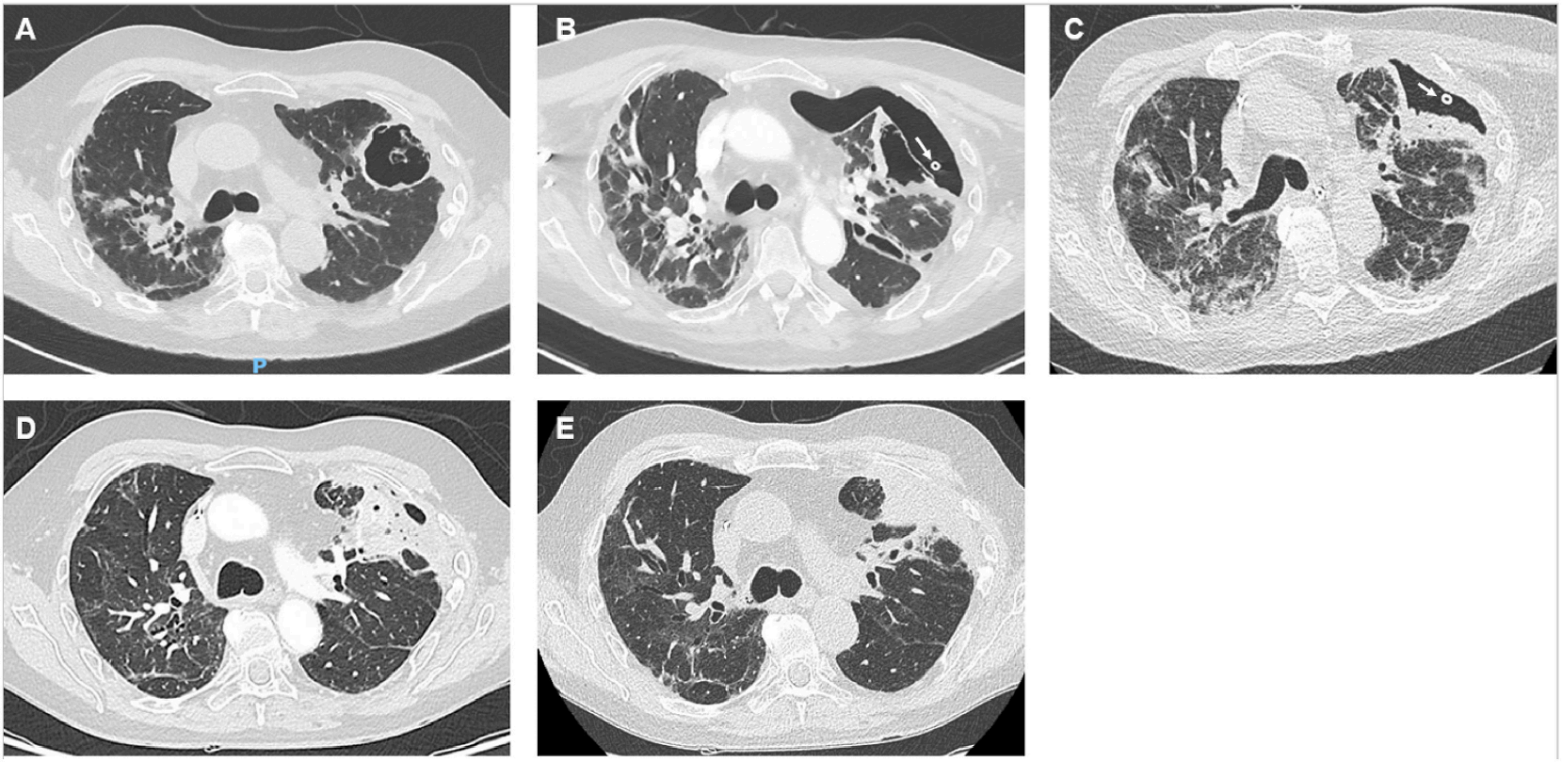
CT scan showing A: Aspergillosis subpleural cavitation before pneumothorax; B: left pneumothorax with alveolar-pleural fistula resulting from the rupture of the pulmonary cavitation (white arrow indicates the cross-section of the drain catheter); C: left lung expansion after EBV placement (white arrow indicates the cross-section of the drain catheter); D and E: aspergillosis cavitation disappearing giving place to a residual consolidation.

A 62-year-old male experienced *anti*-MDA5 dermatomyositis with lung involvement. Initially, he was treated with 1mg/kg/d of methylprednisolone and tofacitinib. Four months later, due to the lack of disease control in the lungs, he subsequently underwent immunosuppressive treatment, which included a 1-g bolus of methylprednisolone followed by a dosage of 1mg/kg/d, along with tacrolimus and tofacitinib. This treatment was completed with plasma exchanges. Two months after this treatment scale-up, a thoracic CT scan revealed the occurrence of cavitation in the subpleural area of the left upper lobe (Fig. 1A). The next month, the patient's clinical condition worsened suddenly. Acute respiratory failure, marked by tachypnoea and hypoxia requiring oxygen therapy up to 12 L per min delivered with high-concentration mask, justified patient admission to the critical care unit. First clinical examination and chest X-ray revealed a complete left pneumothorax. A 14-French “pig's tail” drain catheter was inserted in pleural cavity by Seldinger technique and axillary approach. A CT scan showed an alveolar-pleural fistula that occurred because of the rupture of the pulmonary cavitation (Fig. 1B).

Diagnostic work-up included bronchoscopy and pleural fluid examination. The microbiological evaluation yielded positive culture for *Aspergillus fumigatus* in both bronchoalveolar lavage and pleural fluid, as well as the presence of galactomannan antigen in the bronchioloalveolar fluid, however, the serum tested negative. The anti-*Aspergillus* IgG serology was positive, with an index of IE > 80 UA/mL and a positive Western Blot (LDBio Diagnostic, Lyon, France). In total, chronic cavitary pulmonary aspergillosis was diagnosed. Drug susceptibility testing showed a resistance to itraconazole and posaconazole despite susceptibility to voriconazole. However, sequencing of the cyp51a gene showed the presence of the TR_34_/L98H alteration responsible for environmental resistance to azoles. Consequently, effective antifungal therapy was initiated, starting with intravenous liposomal amphotericin B.

**Fig. 2:**
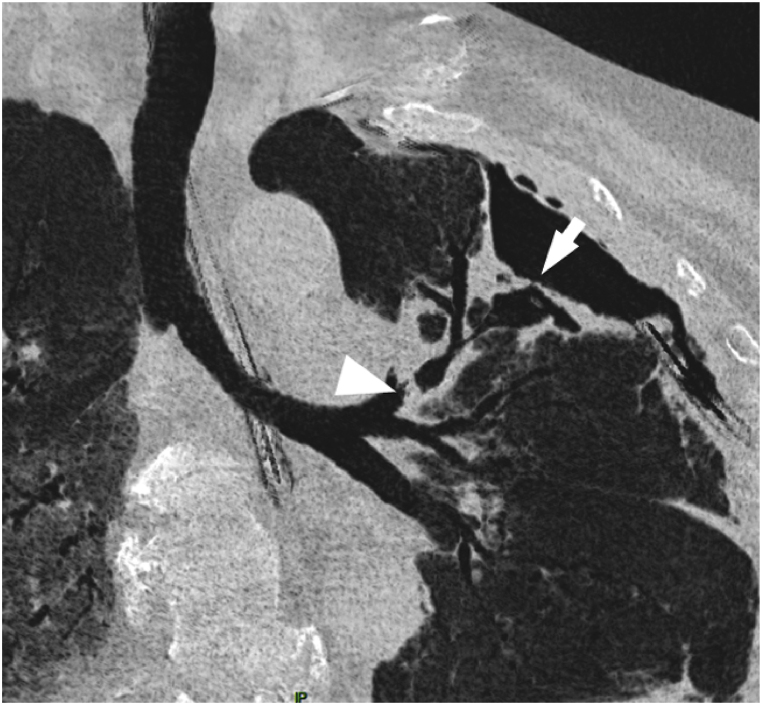
Minimum intensity projection (MinIP) on CT scan revealing alveolar-pleural fistula (White arrow), and the bronchial connection between cavitation and anterior segmental bronchus, where EBV is set (White arrow's head), in the left upper lobe.

**Fig. 3:**
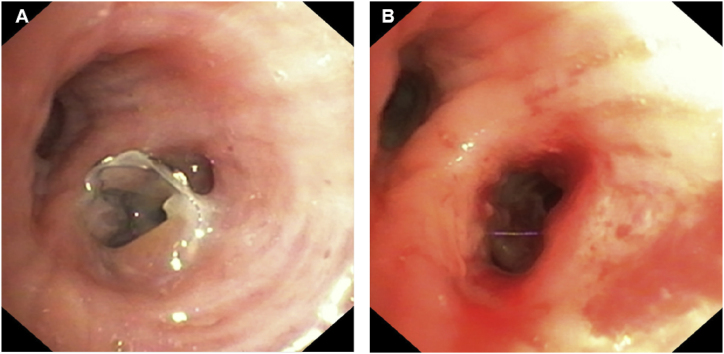
Flexible bronchoscopy view of A: EBV deployed into the anterior segmental bronchus of the left upper lobe; B: limited granulation and inflammation after EBV withdrawal.

Since air leak persisted and left lung pneumothorax failure to re-expand in chest X-ray, physicians conclude to pleural drainage failure after four-week. Patient's clinical condition precluded the possibility of surgical intervention. The examination of the CT scan bronchogram revealed a bronchial connection between the cavitation and the anterior segmental bronchus of the left upper lobe (Fig. 1, Fig. 2). One month after treatment initiation, a single 5.5 mm EBV (Endobronchial Valve Therapy) (Zephyr 5.5®, PulmonX Inc. Neuchatel, Switzerland) was inserted into the anterior segmental bronchus, after the inflation of a balloon catheter which interrupted air leak, during a flexible bronchoscopy performed under general anesthesia. Air leak cessation was promptly observed during the procedure. The EBV was well tolerated. The removal of the thoracic drain taking place two weeks later, following the complete expansion of the left lung (Fig. 1C). Two month after EBV insertion, the cavitation caused by aspergillosis nearly disappeared and was replaced by consolidation (Fig. 1D). The EBV was effectively removed during a flexible bronchoscopy performed under general anesthesia (Fig. 3). One month after the removal of the EBV, the aspergillosis cavitation completely disappeared and was replaced by a residual consolidation (Fig. 1E).

In parallel, antifungal therapy was modified. Intravenous liposomal amphotericin B was switched to intravenous caspofungin four months after antifungal treatment initiation, due to kidney impairment. Finally, oral olorofim, a new antifungal therapeutic class targeting the fungal dihydroorotate dehydrogenase enzyme, was initiated seven months after treatment initiation, because of recurrent central venous catheter infections. Despite the treatment, the anti-*Aspergillus* levels remained elevated, with an index of IE > 80 UA/mL and a positive Western Blot. The residual lesions caused by aspergillosis were successfully treated through surgical resection five month after EBV removal. Pathological examination of the resection specimen revealed aspecific inflammatory granulation tissue rich in neutrophils. Maintenance antifungal therapy was continued by olorofim. The patient passed away eight months after surgical resection due to an unknown lung disease while traveling to his country of birth.

## 3 Discussion

We report a case emphasizing the benefits of EBV for management of bronchopleural fistula related to chronic cavitary pulmonary aspergillosis.

Bronchopleural fistula, characterized by an anomalous communication between the lower respiratory tract and the pleural cavity, represents a common complication of lung surgery and a cause of pneumothorax. Within this framework, bronchopleural fistula presents with persistent air leakage, resulting in heightened patient morbidity, prolonged hospitalization, and increased mortality. In post operative bronchopleural fistula, a retrospective cohort study [9] and a literature review [10], revealed an improvement in air leakage in 93 % of cases (with complete resolution achieved in 48 %) when EBV is used. Despite the absence of randomized controlled trials evaluating the benefits of EBV in bronchopleural fistula, the utilization of EBV could serve as a viable alternative to surgical management [11]. Particularly in the context of pulmonary infections, the potential application of EBV is intriguing given that traditional surgical interventions are often contraindicated. However, the introduction of foreign bodies like EBV may raise concerns in cases of active infection. Several case studies have validated the safety of EBV and its effectiveness in the course of pulmonary tuberculosis and non-tuberculosis mycobacterial lung diseases. Regarding Aspergillus-related lung conditions, the spectrum of the disease ranges widely from allergic manifestations such as allergic bronchopulmonary aspergillosis to more severe invasive infections like invasive aspergillosis. Few case reports have reported the benefits of EBV in managing air leaks in pneumothorax complicating invasive aspergillosis [12], chronic pulmonary aspergillosis [13], and allergic bronchopulmonary aspergillosis [14]. In the case described by Tsim et al. [12], the patient expected EBV three weeks post its placement. In our case study, the insertion of EBV was well tolerated, and its removal was scheduled after two months using flexible bronchoscopy without any complications. The prolonged duration of EBV therapy enabled us to evaluate its potential benefits in chronic pulmonary aspergillosis during the initial stages of antifungal treatment. The response of chronic pulmonary aspergillosis on CT imaging was notably rapid in this case, as evidenced by the disappearance of cavitation after one month with EBV, a stark contrast to the usual 3–6 months timeframe observed with antifungal therapy alone, as reported by Godet et al. [15].

Cavity aeration leads to an augmentation in the local level of oxygen, thereby promoting the proliferation of tuberculosis and non-tuberculosis mycobacteria [16]. This oxygen-mediated enhancement is similarly evident in fungal growth [17] and could conceivably exert an influence on the course of chronic cavitary pulmonary aspergillosis. Furthermore, cavities pose a challenge to treatment efficacy due to limited diffusion of antimicrobial drugs and impaired access to the immune system [16]. As a result, treatment efficacy is significantly reduced in cavitary infections compared to other types of infections, leading to a higher frequency of relapse in both aspergillosis [5] and mycobacterial infections [18].

The principle behind EBV is to seal the drainage bronchus with a one-way valve, allowing air to exit but not enter. This promotes volume reduction in the corresponding lung tissue without affecting the drainage of secretions. Although EBV is an emerging technology, only a few studies on its use in cavitary infection have been found in the PubMed database, but none concerned aspergillosis. Among these studies, only three have reported on the safety and efficacy of EBV in the treatment of pulmonary cavities in patients with multidrug-resistant tuberculosis. In 2016, Corbetta et al. successfully treated cavitary TB using EBV to induce lobar volume reduction. They observed complete collapse of the cavities in four out of five patients without any severe short-term or long-term complications, suggesting that EBV may be a potential treatment for cavities in patients with multidrug-resistant tuberculosis [19]. This conclusion was confirmed in a randomized controlled trial [20]. More recently, An et al. reported a closure rate of 69 % and a culture conversion rate of 100 % in 35 patients with multidrug-resistant tuberculosis [21].

Given the global emergence of chronic cavitary pulmonary aspergillosis [[1], [2], [3], [4]] and the limited effectiveness of current standard treatments, particularly in patients with cavitation [[5], [6], [7], [8]], it is necessary to consider supportive therapies. The case presented demonstrates the EBV effectiveness in closing bronchopleural fistula related to chronic pulmonary aspergillosis and its safety over a 2-month timeframe. Further investigation is warranted to explore the potential benefits of EBV in enhancing the response to antifungal therapy.

## Funding

No financial support was provided for this case report.

## CRediT authorship contribution statement

**Thomas Maitre:** Writing – review & editing, Writing – original draft, Visualization, Validation, Investigation, Formal analysis, Data curation, Conceptualization. **Juliette Camuset:** Writing – review & editing, Visualization, Supervision, Investigation, Conceptualization. **Morgane Faure:** Writing – review & editing, Visualization, Validation, Investigation, Data curation. **Christophe Cracco:** Writing – review & editing, Visualization, Validation, Investigation, Data curation. **Georgina Maalouf:** Writing – review & editing, Data curation. **Yves Allenbach:** Writing – review & editing, Visualization, Validation, Data curation. **Matthias Barral:** Writing – review & editing, Visualization, Validation, Data curation. **Arnaud Fekkar:** Writing – review & editing, Visualization, Validation, Data curation. **Mihaela Giol:** Writing – review & editing, Visualization, Validation, Data curation. **Antoine Parrot:** Writing – review & editing, Visualization, Validation, Data curation. **Jacques Cadranel:** Writing – review & editing, Visualization, Validation, Supervision, Investigation, Conceptualization.

## Declaration of competing interest

The authors declare the following financial interests/personal relationships which may be considered as potential competing interests:FAURE Morgane - Support for attending meetings and/or travel by ERS Milano- Astra Zeneca Receipt of equipment, materials, drugs, medical writing, gifts or other services by Lowenstein ventilation.

## References

[1] Bongomin F., Gago S., Oladele R.O., Denning D.W. (2017). Global and multi-national prevalence of fungal diseases-estimate precision. J. Fungi. (Basel).

[2] Maitre T., Cottenet J., Godet C., Roussot A., Abdoul Carime N., Ok V. (août 2021). Chronic pulmonary aspergillosis: prevalence, favouring pulmonary diseases and prognosis. Eur. Respir. J..

[3] Denning D.W., Pleuvry A., Cole D.C. (2011). Global burden of chronic pulmonary aspergillosis as a sequel to pulmonary tuberculosis. Bull World Health Organ. 1 déc.

[4] Campling J., Jones D., Chalmers J., Jiang Q., Vyse A., Madhava H. (2020). Clinical and financial burden of hospitalised community-acquired pneumonia in patients with selected underlying comorbidities in England. BMJ. Open. Respiratory. Res..

[5] Is S, S D, V M, Kt P, An A A C, et al. Efficacy of 12-months oral itraconazole versus 6-months oral itraconazole to prevent relapses of chronic pulmonary aspergillosis: an open-label, randomised controlled trial in India. Lancet Infect. Dis. [Internet]. juill 2022 [cité 12 mars 2023];22(7). Disponible sur: http://pubmed.ncbi.nlm.nih.gov/35429465/. 10.1016/S1473-3099(22)00057-3 35429465

[6] Agarwal R., Vishwanath G., Aggarwal A.N., Garg M., Gupta D., Chakrabarti A. (2013). Itraconazole in chronic cavitary pulmonary aspergillosis: a randomised controlled trial and systematic review of literature. Mycoses.

[7] Cadranel J., Philippe B., Hennequin C., Bergeron A., Bergot E., Bourdin A. (2012). Voriconazole for chronic pulmonary aspergillosis: a prospective multicenter trial. Eur. J. Clin. Microbiol. Infect. Dis..

[8] Camuset J., Nunes H., Dombret M.C., Bergeron A., Henno P., Philippe B. (2007). Treatment of chronic pulmonary aspergillosis by voriconazole in nonimmunocompromised patients. Chest. mai..

[9] Song J.Y., Park S.G., Lee H.Y., Kim S.R., Kim H.G., Shin S.H. (oct 2022). Comparison of clinical outcomes of pulmonary sequestration in adults between surgery and non-surgery groups. J. Thorac. Dis..

[10] Giddings O., Kuhn J., Akulian J. (2014). Endobronchial valve placement for the treatment of bronchopleural fistula: a review of the current literature. Curr. Opin. Pulm Med. juill..

[11] Hance J.M., Martin J.T., Mullett T.W. (nov 2015). Endobronchial valves in the treatment of persistent air leaks. Ann. Thorac. Surg..

[12] Tsim S., Paton L., Nicholson F., Blyth K.G. (2015). Rescue therapy using an endobronchial valve and digital air leak monitoring in Invasive Pulmonary Aspergillosis. Respir. Med. Case Rep..

[13] Williams R., Krishnadas R., Patel B., Jarad N. (2015). Endobronchial valves in the management of bronchial fistulae caused by bronchopulmonary aspergillosis. BMJ Case Rep..

[14] Ramadurai D., DiBardino D.M., Hong G. (2021). Endobronchial valve placement in secondary pneumothorax related to allergic bronchopulmonary aspergillosis. Respir. Med. Case Rep..

[15] Godet C., Laurent F., Bergeron A., Ingrand P., Beigelman-Aubry C., Camara B. (2016). CT imaging assessment of response to treatment in chronic pulmonary aspergillosis. Chest. juill..

[16] Urbanowski M.E., Ordonez A.A., Ruiz-Bedoya C.A., Jain S.K., Bishai W.R. (2020). Cavitary tuberculosis: the gateway of disease transmission. Lancet Infect Dis. juin..

[17] Grahl N., Shepardson K.M., Chung D., Cramer R.A. (2012). Hypoxia and fungal pathogenesis: to air or not to air? Eukaryotic cell. mai..

[18] Kim H.J., Kwak N., Hong H., Kang N., Im Y., Jhun B.W. (2021). BACES score for predicting mortality in nontuberculous mycobacterial pulmonary disease. Am J Respir Crit Care Med. 15 janv.

[19] Corbetta L., Tofani A., Montinaro F., Michieletto L., Ceron L., Moroni C. (2016). Lobar collapse therapy using endobronchial valves as a new complementary approach to treat cavities in multidrug-resistant tuberculosis and difficult-to-treat tuberculosis: a case series. Respiration.

[20] Levin A., Sklyuev S., Felker I., Tceymach E., Krasnov D. (2016). Endobronchial valve treatment of destructive multidrug-resistant tuberculosis. Int. J. Tubercul. Lung Dis..

[21] An H., Liu X., Wang T., Liu L., Yan M., Xu J. (2022). Endobronchial valve treatment of tuberculous cavities in patients with multidrug-resistant pulmonary tuberculosis: a randomized clinical study. Pathogens. 10 août.

